# Establishment of animal model for the analysis of cancer cell metastasis during radiotherapy

**DOI:** 10.1186/1748-717X-7-153

**Published:** 2012-09-11

**Authors:** Jong Kuk Park, Su Jin Jang, Sung Wook Kang, Sunhoo Park, Sang-Gu Hwang, Wun-Jae Kim, Joo Hyun Kang, Hong-Duck Um

**Affiliations:** 1 Division of Radiation Cancer Biology, Korea Institute of Radiological and Medical Sciences, 215-4, Gongneung-Dong, Nowon-Gu, Seoul 139-706, Republic of Korea; 2 Molecular Imaging Research Center, Korea Institute of Radiological and Medical Sciences, 215-4, Gongneung-Dong, Nowon-Gu, Seoul 139-706, Republic of Korea; 3 Laboratory of Radiation Pathology, Korea Institute of Radiological and Medical Sciences, 215-4, Gongneung-Dong, Nowon-Gu, Seoul 139-706, Republic of Korea; 4 Department of Urology, College of Medicine, Chungbuk National University, Chungbuk, Korea

**Keywords:** γ-Ionizing Radiation, Cancer, Metastasis, Epithelial-mesenchymal transition, Bioluminescence imaging, Animal model

## Abstract

**Background:**

Γ-Ionizing radiation (IR) therapy is one of major therapeutic tools in cancer treatment. Nevertheless, γ-IR therapy failed due to occurrence of metastasis, which constitutes a significant obstacle in cancer treatment. The main aim of this investigation was to construct animal model which present metastasis during radiotherapy in a mouse system *in vivo* and establishes the molecular mechanisms involved.

**Materials and methods:**

The C6L transfectant cell line expressing firefly luciferase (fLuc) was treated with γ-IR, followed by immunoblotting, zymography and invasion assay *in vitro.* We additionally employed the C6L transfectant cell line to construct xenografts in nude mice, which were irradiated with γ-IR. Irradiated xenograft-containing mice were analyzed via survival curves, measurement of tumor size, and bioluminescence imaging *in vivo* and *ex vivo*. Metastatic lesions in organs of mice were further assessed using RT-PCR, H & E staining and immunohistochemistry.

**Results:**

γ-IR treatment of C6L cells induced epithelial-mesenchymal transition (EMT) and increased cell invasion. In irradiated xenograft-containing mice, tumor sizes were decreased dramatically and survival rates extended. Almost all non-irradiated xenograft-containing control mice had died within 4 weeks. However, we also observed luminescence signals in about 22.5% of γ-IR-treated mice. Intestines or lungs of mice displaying luminescence signals contained several lesions, which expressed the fLuc gene and presented histological features of cancer tissues as well as expression of EMT markers.

**Conclusions:**

These findings collectively indicate that occurrences of metastases during γ-IR treatment accompanied induction of EMT markers, including increased MMP activity. Establishment of a murine metastasis model during γ-IR treatment should aid in drug development against cancer metastasis and increase our understanding of the mechanisms underlying the metastatic process.

## Background

Metastasis is one of the distinct characteristics of cancer and presents a major obstacle to cancer treatment. An estimated 50% of all cancer patients develop metastasis, which is an important limiting factor in establishing a cure, and elucidation of the mechanisms underlying metastasis is thus important to overcome failure of treatment [1]. Induction of metastasis is critically dependent on the capability of cancer cells to mobilize from the original site. Cancer cells acquiring motility access the vasculature of the lymphatic system via invasion, and attach and proliferate to distant sites with a favorable microenvironment [1-3]. Metastasis is accomplished when proliferating cancer cells in secondary sites form another cell mass that may display different characteristics, in particular, drug or radioresistance, from the original cancer, as a consequence of encountering various microenvironmental and stress factors distinct from the primary site [4-6]. Therefore, migrating cancer cells in metastases could be resistant against cell death, and the resistance might depend on activation of the phosphatidylinositol 3-kinase (PI3Kinase) signaling pathway. Activation of the PI3Kinase pathway in metastatic cancer cells due to the highly glycolytic state of the cells [7,8] and aerobic glycolysis could also induce drug resistance in cancer cells [9,10]. Resistance against anoikis induced upon cell detachment from the extracellular matrix (ECM), might also be involved in survival of migrating cancer cells [11,12]. A few reports have suggested that induction of resistance against anoikis is also derived from activation of the PI3Kinase signaling and extracellular signaling-receptor kinase (ERK) via various proteins involving TrkB, its ligand brain-derived neurotrophic factor (BDNF), and hepatocyte growth factor (HGF) [13,14]. PI3Kinase and ERK may also participate in cancer cell migration and invasion by activating various ECM-degrading enzymes, such as the matrix metalloproteinase (MMP) family proteins [15-17]. MMP proteins represent one of the major markers of epithelial-mesenchymal transition (EMT) as well as metastasis [18]. Recent studies have shown that EMT is a fundamental cellular mechanism, promoting cell migration and loss of cell polarity during organ formation and differentiation. Development of gastrulation, neural systems and various internal organs, such as pancreas and liver, are required from induction of EMT [19,20]. In cancer, EMT plays various roles in the maintenance of cancer stemness and induction of metastasis, and is inducible by different growth factors, hormones, and intracellular molecules. Several regulators, such as Snail, Twist1 and SIP1, have been shown to mediate EMT and metastasis under signaling of hypoxia/HIF-1, Wnt, Notch, and TGF-β. Environmental factors, including nicotine, ultraviolet light (UV) and γ-IR, also promote EMT [20-22]. Induction of EMT appears to be related to resistance against chemotherapy reagents, such as tamoxifen and gemcitabine, as well as radiotherapy [23-25]. Furthermore, EMT stimulates acquisition of elongated cancer cell survival during movement from the primary cancer to distal metastasis site [26].

Around 50% of all solid cancer patients receive radiation therapy, one of the major current treatment methods. However, recent reports have demonstrated that γ-IR induces an increase in invasiveness of several cancer cell types, including glioma, hepatocellular carcinoma, and lung cancer cells [22,27,28]. This increase in invasiveness is accomplished via enhanced activity and expression of MMP family proteins promoted by various intracellular pro-survival signaling pathways, such as NF-κB and PI3-kinase/AKT. These proteins are known cell survival factors that endow resistance against various stress conditions [29-32].

In the present study, we attempted to establish whether γ-IR-induced invasion and metastasis are stimulated in our *in vitro* C6L cell line and *in vivo* systems, and further identify the associated changes in signal pathways or mice physiology. We constructed an animal model system with a view to clarifying the intracellular molecular events underlying the promotion of metastasis after γ-IR treatment for primary cancer and developing effective anti-metastatic reagents. Our results demonstrate that γ-IR treatment of cancer cell lines and mice xenografts triggers invasion and metastasis. In particular, γ-IR-treated cancer cells or mouse xenografts and metastatic lesions in mice bearing γ-IR-treated xenografts also display typical EMT marker expression patterns, such as increased vimentin or MMP-2 expression, decreased E-cadherin, and enhanced activity of MMP-2. Our results collectively suggest that γ-IR-induced invasion or metastasis results from induction of EMT, and inhibition of EMT may thus be a means to enhance the effectiveness of radiation therapy.

## Materials and methods

### Cell culture and irradiation of γ-IR

Rat glioma cell C6 cells were obtained from American Type Culture Collection (Rockville, MD), and grown in DMEM (Invitrogen, Grand Island, NY) supplemented with 10% fetal bovine serum (Invitrogen) in a humidified 5% CO_2_ incubator at 37°C. We employed C6 to construct C6L transfectant cells containing the firefly luciferase (fLuc) gene in lentiviral vectors and selected with blastidin treatment (5 mg/mL). Irradiation with various doses of γ-IR was performed with a γ-IR irradiator using ^137^Cs as a source of γ-rays (Atomic Energy of Canada, Ltd., Mississauga, ON).

### PCR analysis

Total RNA was isolated from metastatic lesions, and used as a template to produce cDNA using SuperScript III First-Strand Synthesis for RT-PCR (Invitrogen, CA). Synthesized cDNA was amplified using *Taq* DNA polymerase (iNtRON, Seoul) with the following primers for *fLuc*: forward, 5’- CGC CTT GAT TGA CAA GGA TGG, and reverse, 5’- GGC CTT TAT GAG GAT CTC TCT. The forward primer used for *glyceraldehyde-3- phosphate dehydrogenase* (*GADPH*) was 5’- GGT GAA GGT CGG TGT GAA CG and the reverse primer was 5’- CTC GCT CCT GGA AGA TGG TG.

### Invasion analysis

C6L cells were seeded in a 35 mm cell culture dish and irradiated with 1, 3, 5, or 7 Gy of γ-IR. Transwell systems containing a 0.8 μm pore (Corning, NY) were coated with Matrigel (1 mg/mL, Invitrogen), and pre-irradiated cells (2 × 10^4^) were washed with serum-free media twice after 18 h of irradiation. Cells were added to the upper chamber and serum-free medium containing 0.1% BSA added to the lower chamber of each transwell, and incubated for 18 h with 5% CO_2_ at 37°C. Cell staining was performed with deep quick solution (Merck, Whitehouse Station, NJ), according to the manufacturer’s protocol. Photographs of microscopic images of stained cells were taken under a microscope and counted, and then statistical analyses were performed.

### Immunoblot analysis

C6L cells were seeded in a 60 mm cell culture dish and irradiated with 3 Gy of γ-IR. Irradiated cells were trypsinized and washed with 1× ice-cold PBS. RIPA buffer [50 mmol/L Tris, pH.8.0, 150 mmol/L NaCl, 1% NP-40, 0.5% deoxycholic acid, and 0.1% sodium dodecyl sulfate (SDS)] containing protease and phosphatase inhibitor cocktail (Sigma) was used to dissolve harvested cell pellets for acquiring whole-cell protein lysates. Cell lysates were separated by 12% SDS-polyacrylamide gel electrophoresis (PAGE) and transferred to nitrocellulose membranes (Invitrogen Co., Carlsbad, CA). Protein-transferred membranes were incubated with primary antibodies against E-cadherin, MMP-2 (Santa Cruz Biotechnology, Santa Cruz, CA) or Vimentin (Dako, Glostrup, Denmark). Primary antibody-attached membranes were washed with PBS-Tween 20 (PBS-T) and incubated with the appropriate secondary antibody. A chemiluminescence kit (Thermo scientific, Rockford, IL) was used to detect target proteins on the nitrocellulose membrane.

### Gelatin zymography

C6L cells were irradiated with 3 Gy, and the media of irradiated cells replaced with serum-free medium. Cells were incubated for 24 h in serum-free medium under a humidified atmosphere with 5% CO_2_ at 37°C, collected and loaded on to a 0.1% gelatin SDS-PAGE gel (10%). After separation of proteins, gel renaturation was performed with 2.5% Triton X-100 buffer for 1 h, and the gelatin gel incubated with developing solution (50 mM Tris pH 7.5, 20 mM NaCl, 10 mM CaCl_2_, 1 μM ZnCl_2_, 0.1% NaN_3_) for 24 h at 37°C, followed by staining with 5% Coomassie Brilliant Blue (Sigma, St. Louis, MO).

### Propidium iodide (PI) uptake analysis

A PI uptake analysis was performed, as described in our previous report [33], to detect γ-IR-induced cell death on C6L cells. C6L cell samples were prepared as for the invasion analysis; C6L cells were seeded in a 35-mm cell culture dish and cultured overnight. The seeded cells were irradiated with 1, 3, 5, 7 Gy of γ-IR, and then incubated for an additional 18 hours. The cells were harvested and stained with PI solution (5 mg/mL, Sigma, St. Louis, MO, USA), and then analyzed with a BD FACSCalibur flow cytometer (BD Biosciences, San Jose, CA, USA).

### Formation of Xenografts and Irradiation with γ-ionizing radiation

C6L cells (5 × 10^5^) were injected s.c. into the right hind legs of 6 week-old BALB/cAnNCrj-nu/nu mice (Charles River Japan, Inc., Tokyo) to construct xenografts, as described in a previous report by Park *et al.*[34]. Xenografts reaching more than 100 mm^3^ were treated with γ-IR at 10 Gy per day for 5 days, but not the mock control group. Mice were anesthetized i.p. with Zoletil 50™ (VIRBAC Laboratories, Carros) and fixed on an acryl plate. Xenografts were locally irradiated with a ^60^Co γ-IR source (Theratrom 780; AECL, Ltd., Mississauga, ON), while other body parts were protected with lead blocks. Tumor sizes and survival curves of the control or radiation-treated groups were assessed for 62 days. Tumor sizes were established with a caliper rule (Mitutoyo Co. Japan), and the volume of each xenograft calculated as follows: [(short axis^2^ × long axis)/2].

### Bioluminescence imaging acquisition

Bioluminescence imaging was performed with a CCD camera mounted in a light-tight specimen chamber (IVIS200, Xenogen, CA). For *in vivo* imaging, mice were administered 100 μL of 2.5 mg/100 μL D-luciferin potassium salt i.p. and anesthetized with 2% isoflurane. Imaging and quantification of signals were controlled with the acquisition and analysis software Living Image V. 2.50 (Xenogen), as described previously by Jang *et al.*[35].

### Histological analysis and Immunohistochemistry

To perform histological analysis, metastatic lesions were fixed with formaldehyde and embedded in a paraffin block. Sliced tissues were stained Hematoxylin & Eosin solution, and histological analysis performed under a microscope. Immunohistochemistry for the detection of EMT markers in lesions was also performed with the CAP-PLUS™ Broad Spectrum kit (Zymed Laboratories Inc. South San Francisco, CA), as described in the manufacturer's protocol. Tissue sections of the lesions were deparaffinized with xylene, rehydrated, and incubated in citric acid buffer (0.01 M, pH 6.0) for 20 min at 100°C. Incubated sections were cooled slowly at room temperature for 20 min, and endogenous peroxidase activity blocked by treatment with H_2_O_2_ for 15 min. Sections were incubated with primary antibodies (anti-Vimentin and anti-E-cadherin) overnight at 4°C and washed with 0.05% Tween 20-containing PBS buffer three times. Secondary antibody, streptavidin, and DAB were sequentially added to the sections for visualization of vimentin and E-cadherin, followed by treatment with autohematoxylin for counterstaining of nuclei.

### Statistical analysis

**Figure 1:**
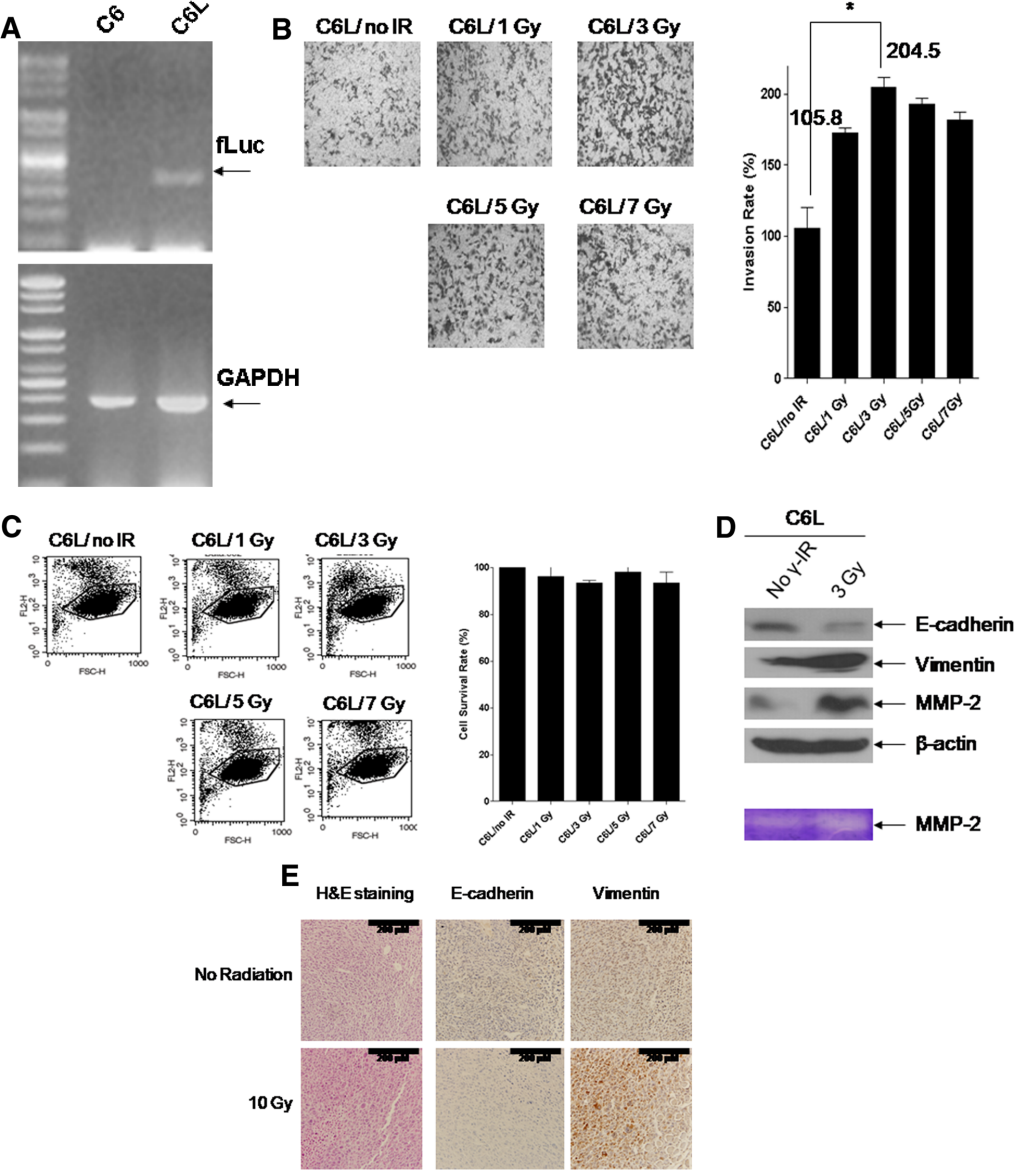
**Γ-IR promotes invasion of C6L cells through induction of EMT.** (**A**) C6L, stably transfected cells containing the firefly luciferase gene, was constructed from Rat glioma C6 cells. (**B**) C6L transfectants were seeded and treated with 1, 3, 5, or 7 Gy of γ-IR, and the invasion assay performed as described in Materials and methods. C6L/no IR represents untreated mock control and C6L/1, 3, 5, or 7 Gy the γ-IR-treated groups. The *p* values of the C6L/no IR and C6L/3 Gy groups were determined with the t-test. **: *p* < 0.01 (*p* value = 0.001). (**C**) Propidium iodide (PI) uptake analysis. C6L/no IR represents the γ-IR-untreated mock control, and C6L/1, 3, 5, or 7 Gy represents the γ-IR-treated groups. (**D**) C6L transfectants were treated with 3 Gy of γ-IR, and immunoblot and gelatin zymography analyses performed. No γ-IR represents untreated mock control and 3 Gy signifies the γ-IR–treated groups. (**E**) Treatment of xenografts with γ-IR also induced EMT, which was detected with IHC (200×). No γ-IR indicates γ-IR-untreated mock control and 10 Gy is the γ-IR-treated group. Γ-IR treatment (10 Gy) was performed as described in Figure 2A, and the tissues collected on day 35 after injection of C6L.

All experiments were repeated at least three times, but Figures 1B and 1E were performed at duplicate. Data were calculated with GraphPad Prism (GraphPad Software INC. CA), and differences between the experimental groups determined using a *t*-test and one way-ANOVA. Error bars indicate standard deviations (SD), and *p*-values were calculated.

## Results

### Γ-IR promotes invasiveness via induction of EMT *in vitro* and *in vivo*

In this study, we investigated the possibility that γ-IR promotes metastasis in an *in vivo* murine model system. We constructed a firefly luciferase (fLuc)-expressing C6L transfectant cell line derived from the rat glioma cell line, C6. Construction of the C6L cell line was confirmed with PCR analysis using *firefly luciferase* primer and *GAPDH* (Figure 1A). We observed expression of exogenous *fLuc* gene in the C6L cell line, but no detectable *fLuc* gene in C6 cells. This result implies that expression of fLuc is a prominent selective marker in the mouse system *in vivo* in addition to bioluminescent assay *in vitro*. Enhanced invasion of C6L cells following γ-IR treatment was detected in a dose-dependent manner based on quantitative analysis using matrigel-coated transwells (Figure 1B). Treatment with 3 Gy of γ-IR induced the maximum increase in C6L cell invasion by more than two-fold. But treatments of γ-IR in a dose-dependent manner did not affect C6L cell survival (Figure 1C). To further elucidate the intracellular signaling machinery underlying the γ-IR-induced increase in the invasive ability of C6L, we performed immunoblot analysis with the EMT markers, E-cadherin, vimentin, and MMP-2 (Figure 1C upper panel). We detected decreased E-cadherin expression and increased vimentin/MMP-2 expression, concurrent with the typical induction patterns of EMT markers. Increased MMP-2 activity was also observed with the gelatin zymography assay (Figure 1C, lower panel). In another preliminary experiment, we treated xenografts with 10 Gy of γ-IR, and isolated the tissue for IHC analyses with EMT markers (Figure 1D). IHC analysis with anti-E-cadherin or anti-vimentin antibodies revealed a decrease in E-cadherin and increase in vimentin expression, respectively. Based on these findings, we propose that induction of EMT might be the underlying cause of γ-IR-induced metastasis.

### Γ-IR caused regression of tumors and prolonged survival but did not block death of the *in vivo* model perfectly

**Figure 2:**
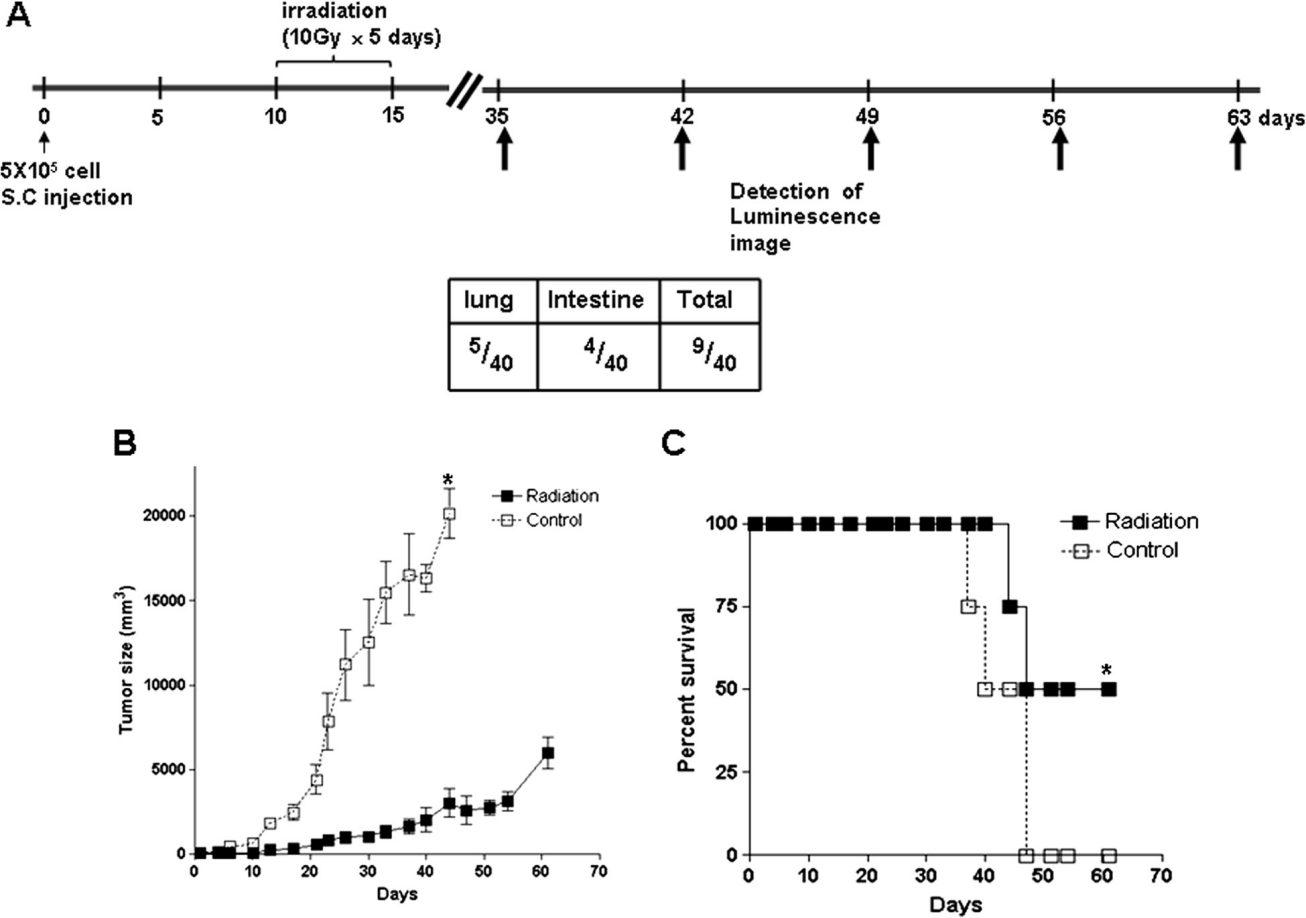
**Treatment with γ-IR affects tumor size and percentage survival in mice.** (**A**) Radiation treatment schedule (scheme, upper part) and occurrence of metastasis detected with bioluminescence imaging (box, lower part). (**B**) Detection of tumor sizes. Radiation (black square) indicates the γ-IR-treated group (*n* = 5) and control (white square) signifies the control group (*n* = 4). Tumor sizes of mice were detected as long and short axis lengths, and calculated as follows: (short axis length^2^ × long axis length)/2. The *p* values between γ-IR-treated and control groups were calculated with the t-test. *: *p* < 0.01 (*p* value = 0.0014). (**C**) Survival rates of mice. Survival rate was calculated as a percentage of each group. The *p* values between γ-IR-treated and control groups were obtained with the t-test. *: *p* < 0.01 (*p* value = 0.0063).

To further establish whether γ-IR induces metastasis of cancer *in vivo*, we constructed xenografts with 5 × 10^5^ C6L cells irradiated with γ-IR, as described in Materials and methods. The experimental schedule in which bioluminescence images were detected every 7 days from day 35 is presented in Figure 2A. Preliminary studies on xenograft size and survival in mice subjected to γ-IR treatment were performed (Figures 1A and 2B). We calculated the time-periods needed for xenograft sizes of each group to reach 2500 mm^3^ (Figure 2B). Notably, the control group reached this size in 4 days and the γ-IR treatment group in 45.7 days, revealing a 41.3-day growth delay in terms of xenograft size (Figure 2B). With regard to survival rates, 50% of γ-IR-treated mice survived until day 61, while control mice did not survive for more than 44 days (Figure 2C). Our results indicate that a total of 50 Gy of γ-IR effectively reduces xenograft size and extends survival in mice, but does not eliminate the tumor completely. Failure of complete tumor eradication by γ-IR suggests the possibility of tumor recurrence. As shown in Figures 2B and 2C, rapid tumor growth and death of mice in the γ-IR treatment group were detected from day 40. Death of mice displaying smaller tumor sizes in the γ-IR treatment group, compared to those in the control group indicates the occurrence of metastasis at a secondary site.

### Metastases following γ-IR treatment were detected in the murine *in vivo* model

**Figure 3:**
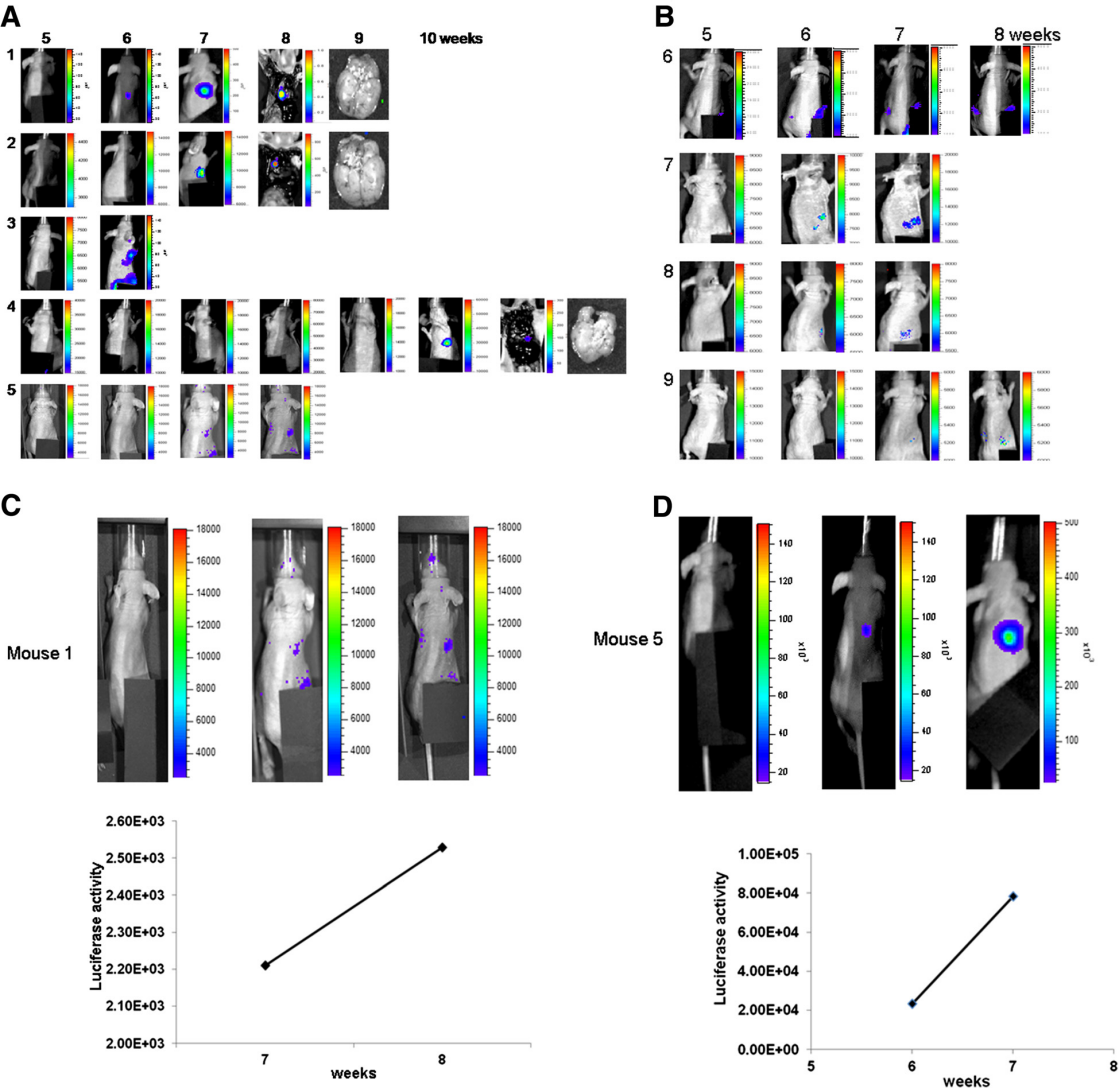
**Detection of metastasis of γ-IR-treated mice using bioluminescence imaging.** Xenograft formation and γ-IR treatment were performed as described in Materials and methods. Images were detected over a week between weeks 3 and 9 of radiation treatment. Bioluminescence imaging of metastasis was performed after mice were anesthetized with isoflurane in a live state. (**A**) Bioluminescence images of metastasis in the lungs. The upper Figure represents the schedule of bioluminescence imaging and lower box indicates the number of metastases and total mice. Bioluminescence images of metastasis in the lung. The horizontal number indicates the week and the vertical number represents each mouse. *Ex vivo* images including brains were also detected in the last week (mouse numbers 1, 2, and 4). (**B**) Bioluminescence images of metastasis in the intestine. The horizontal number indicates the week and the vertical number represents each mouse. (**C** and **D**) Relationship analysis of fLuc activity and tumor volume of mice (numbers 1 and 5) in the lung metastasis group.

Metastasis from the primary site to internal organs following γ-IR treatment was detected with bioluminescence imaging *in vivo* and *ex vivo* using fLuc activity of C6L cells in xenografts. We constructed xenografts of 40 mice in total and treated them with γ-IR, as shown in Figure 2A. Bioluminescence images were detected, as described in Materials and methods. Bioluminescence signals in the body trunks of mice, except the xenograft sites, were observed in 9 (22.5%) among the 40 mice. Specifically, 5 mice displaying bioluminescence presented signals in the chest region and 4 in the abdomen region (Figures 3A and 3B). To ascertain the occurrence of metastasis and the exact site, mice were sacrificed, and bioluminescence imaging (*ex vivo* bioluminescence image) was performed again with the abdomen open to obtain the precise location of tumor growth in organs including brains. *Ex vivo* bioluminescence images were detected in 3 among 5 mice displaying signals in the chest region (Figure 3A). Lung metastases were observed in *ex vivo* bioluminescence images, but brain metastases were not. Relationship analysis of fLuc activity with tumor volume *in vivo* indicated a time-dependent increase in signal intensity (Figures 3C and 3D).

### Metastases in the animal model may be accompanied by EMT induction

**Figure 4:**
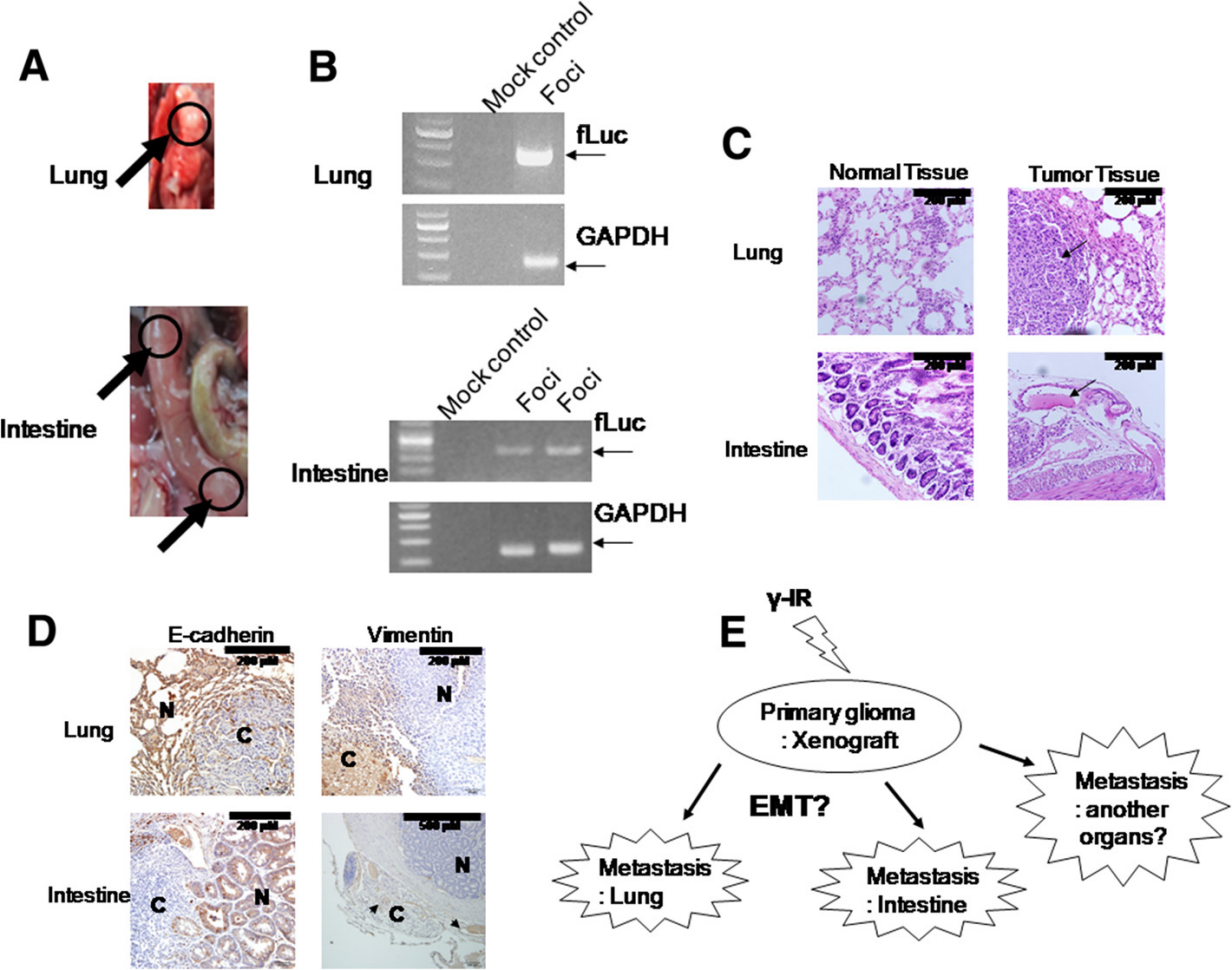
**Histological or genetic analysis of metastatic lesions.** (**A**) Mice displaying bioluminescence signals upon imaging were scarified and dissected. Lesions in the lung and intestine were observed (black arrows and circles). (**B**) The fLuc gene in lesions was detected with PCR. (**C**) Histological analysis revealed that each lesion displays characteristics typical of cancer tissue (200×). (**D**) IHC analysis. E-cadherin was expressed in normal tissue, while vimentin was expressed in cancer tissue (N: normal tissue, C: cancer tissue) [200× except to vimentin staining of intestine (100×)]. (**E**) Scheme of γ-IR – induced metastasis.

Although *ex vivo* bioluminescence image analysis failed to completely reveal bioluminescence signals in mice subjected to whole autopsy, several lesions estimated as neoplasms in the lung and intestine were observed with visual assessment (Figure 4A). Lesions were further analyzed by PCR with *fLuc* or *GAPDH* primers (Figure 4B) and histological experiments (Figure 4C). As shown in Figure 4B, each lesion expressed the fLuc gene, indicative of its origin from C6L xenografts as a primary cancer. H & E staining in histological analysis revealed the formation of solid tumors in normal lung and intestine tissues (Figure 4C). These results suggest that γ-IR promotes metastasis *in vivo* as well as *in vitro*, and induction of cancer spread by irradiation is accomplished randomly. To identify changes in intracellular signaling or physiological phenomena occurring in γ-IR-induced metastasis, we assessed the expression of ECM markers in histological tissue samples (Figure 4D). IHC analysis with E-cadherin or vimentin disclosed different expression patterns in cancer and normal sites. Specifically, E-cadherin was expressed at the normal site in each tissue (lung and intestine), while vimentin was expressed at the cancer site in each tissue, especially in the intestine, where expression was detected in the lesion center.

## Discussion

Although γ-IR is one of most useful known tools for cancer treatment to date, many investigators have reported metastasis from primary cancer following irradiation. As reported previously in a review by C.F. von Essen [36], various researchers have described two distinct cases of metastasis induced by γ-IR, whereby (1) increased metastasis is triggered following local irradiation against primary cancer, and (2) localization of metastases is increased in γ-IR-pretreated normal tissues. In the current study, we have demonstrated that γ-IR treatment of primary cancer xenografts of the C6L cell line induces distant metastasis *in vivo* and enhances the invasiveness of C6L *in vitro* via induction of EMT. We generated a C6L cell line expressing fLuc from parental C6 and further examined whether local γ-IR treatment of xenografts constructed from our cell line promotes distant metastasis. In our previous report [35], we used C6 cells to construct C6TL cells containing the herpes simplex virus type-1 thymidine kinase (HSV1-tk) and firefly luciferase (fLuc) genes for gene therapy against glioma and found that the genetically modified C6 cell line is a very effective monitoring system for Bioluminescence imaging or microPET due to their high fLuc activity and iodine-125 iodovinyldeoxyuridine uptake. We used nude mice, because the entire body of hairless nude mice provided a chance to observe the Bioluminescence signal more efficiently in the body trunk. C6L was initially generated from the C6 rat glioma cell line derived from C6 glioblastoma tumors in Sprague–Dawley and Wistar rats, which display significant invasion and a diffuse infiltrating border in which each C6 cell moves to normal brain tissue beside the tumor area [37]. Thus, C6 could be useful to investigate invasion of glioma as a glioblastoma model system. Park *et al.*[28] demonstrated that γ-IR treatment increases invasion through activity or expression of MMP-2 via activation of Src/epidermal growth factor receptor-mediated p38/Akt and PI3-Kinase/Akt signaling in various non-functional *PTEN*-bearing glioma cells, including C6. We additionally showed that irradiation of the C6L cell line via induction of EMT (Figure 1C). Our results are consistent with previous reports showing that γ-IR-treated non-small cell lung cancer (NSCLC) cell lines display increased invasion via EMT induction, characterized by activation of MMP protein [22,38]. Moreover, we demonstrated previously that γ-IR promotes the level of Bcl-xL, a pro-survival protein, in a manner dependent on signal transducer and activator of transcription 3 (STAT3) phosphorylation [22]. Treatment with γ-IR also enhanced invasion of hepatocellular carcinoma cell (HCC) cell lines via the PI3-Kinase/Akt/NF-κB/MMP-9 pathway [27].

Next, we performed experiments to establish whether the increased invasiveness of C6L by γ-IR promotes metastasis *in vivo*. The schedule for γ-IR treatment of xenografts was followed as described by Camphausen *et al*. [39], who used Lewis lung carcinoma (LLC-LM) cells to confirm that γ-IR promotes metastasis in an animal model and suggested that increased levels of MMP-2 may be related to enhancement of metastases. Survival and tumor sizes in mice showed that local γ-IR treatment decreases tumor size and elongates survival of mice but do not block metastasis completely. C.F. von Essen also suggested three possible mechanisms underlying the promotion of metastases by local irradiation, specifically, (1) direct alterations in γ-IR-treated cancer cells (2) promotion of entry of cancer cells into the circulation, and (3) extension of time needed for entry of cancer cells into the circulation. Data from the present study indicate that γ-IR treatment can elongate survival times in mice, leading to entry of cancer cells into the circulation. Thus, the third hypothesis of C.F. von Essen is consistent with our results. We need to test the other hypotheses in future studies to clarify the molecular mechanism underlying γ-IR-induced metastasis.

To detect the occurrence of metastasis in internal organs of the *in vivo* murine system, we used the bioluminescence imaging system containing charged coupled device (CDD) detectors. As this system provides noninvasive *in vivo* optical images, we traced the occurrence of metastasis during several weeks. Molecular imaging techniques are based on nuclear medicine that is concentrated to manage and diagnose diseases through injection of radiolabeled tracers [40]. As recent development of bioluminescence imaging systems results from advances in molecular biology and biochemistry, the technique consists of optical imaging systems with fluorescence imaging whereby molecular probes are required to detect specific signals in living subjects. Various biocompatible molecular probes, including chemicals and reporter genes, have been used for optical imaging. In particular, several intracellular reporter genes have been developed and applied, including GFP, thymidine kinase, cytosine deaminase, tyrosinase, and luciferase [41]. Luciferases constitute a family of enzymes originating from diverse fireflies or beetles that emit their own specific wavelength of light using specific substrates and biochemical pathways [42]. As shown in Figures 3A and 3B, we detected bioluminescence signals in the chest or abdomen of living mice, which were regarded as metastatic lesions. Some mice showed increased bioluminescence signals in a time-dependent manner in a relationship analysis of fLuc activity. *Ex vivo* image detection of bioluminescence signals at the final step was also performed to confirm the signals in body trunks of mice. Our results provide evidence that optical bioluminescence signal detection is a useful non-invasive diagnostic tool for metastasis.

We observed several metastatic lesions in the lung or intestine after autopsy. Interestingly, metastatic lesions were not detected in several internal organs simultaneously in each mouse, but the possibility that undetected metastatic lesions in other visceral organs were discovered remained, because we did not perform a complete dissection of the entire mouse. Metastatic lesions of the lung were identified with hardened tissues, and those of the intestine formed white, small-sized cysts. RT-PCR results obtained using RNA from metastatic lesion tissues indicated that these lesions contain the fLuc gene and therefore originate from C6L cells in the primary cancer located a distance away. Histological analysis with H & E staining revealed that the metastatic lesions are typical cancerous lesions with poorly differentiated morphology, compared with those of healthy lung and intestine tissue. IHC examination with anti-E-cadherin and anti-vimentin antibodies further showed that cancer sites in metastatic lesions contain low E-cadherin/high vimentin levels, while conversely, normal tissue sites present with high E-cadherin/low vimentin expression levels. These results were inconsistent with the typical EMT theory hypothesizing that increases in cancer cell migration/invasion via induction of EMT promote cancer cell movement to a new site for metastasis formation and that mesenchymal-to-epithelial transition (MET) is required for settlement of cancer cells [19,43]. However, several clinical reports indicate that loss of E-cadherin in gastric/colorectal cancer and increased vimentin expression in NSCLC/gastric cancer are markers for cancer progression, metastasis and poorer prognosis [44-47]. Therefore, we could postulate that MET might be a transient event that is induced to promote formation of the metastasis site, followed by progression of metastatic lesions.

However, there are other possibilities that EMT might not be the main or only cause of radiation-induced metastasis in our system, because Tarin *et al.* reported no confirmative evidence that EMT can be induced *in vivo*[48]. Instead, fused hybrids of macrophages and non-metastatic cancer stem cells can metastasize *in vivo*[49-51]. Moreover, Mor-Vaknin *et al.* reported that activated macrophages induce secretion of vimentin and up-regulate MMP proteins [52]. Because radiation might be a cause of macrophage-cancer cell fusion [53,54], fusion hybrids of macrophages and cancer cells in our system might be discovered. According to Kliopp *et al.* irradiated tumors recruit circulating mesenchymal stem cells into their microenvironment by increasing expression of several cytokines that might activate macrophages [55]. These previous reports suggest that further studies on the relationship between the immune system and radiation-related metastasis are needed to validate our animal model. EMT expression markers at distal metastatic lesions are also required to investigate the relationship with the immune system.

Together, our results suggest that radiotherapy alone could promote metastasis as an undesired effect and γ-IR-induced metastasis *in vivo* is evoked via the EMT pathway. Data obtained with our *in vivo* animal model may be employed for the development of drugs blocking radiation-induced metastasis. In previous reports, administration of recombinant angiostatin after cancer cell injection *in vivo* blocked the formation of metastases by γ-IR [39], and neutralization of TGF-β with its specific antibody inhibited γ-IR-induced metastasis [56]. Although molecular targets for blockage of γ-IR-induced metastases in our system are yet to be identified, the EMT pathway may be a significant candidate for drug development. In fact, upstream molecules of the EMT pathway, including TGF-β receptor, integrin, Wnt, or Notch signaling, represent targets for anti-cancer drug development [20,57]. Development of novel drugs to block γ-IR-induced metastasis may maximize the beneficial effects of radiotherapy and improve treatment outcomes.

## Conclusion

This study is focused on the construction of an animal model for the development of inhibitor to block the metastatic process which occurs during radiotherapy. Γ-IR treatment did not block the occurrence of metastases in mice containing xenografts of C6L cells. Induction of EMT markers was detected in γ-IR – treated cells, xenografts, and metastatic lesions in mice. Therefore, our results also suggested EMT might be one of the major therapeutic targets to block metastasis

## Abbreviations

EMT: Epithelial-mesenchymal transition; ECM: Extracellular matrix; IR: Ionizing radiation; PI3Kinase: Phosphatidylinositol 3-kinase; ERK: Extracellular signaling-receptor kinase; BDNF: Brain-derived neurotrophic factor; HGF: Hepatocyte growth factor; MMP: Matrix metalloproteinase; UV: Ultraviolet light; fLuc: Firefly luciferase; PI: Propidium iodide; SDS: Sodium dodecyl sulfate; PAGE: Polyacrylamide gel electrophoresis; GAPDH: Glyceraldehyde-3- phosphate dehydrogenase; SD: Standard deviations; STAT3: Signal transducer and activator of transcription 3; HCC: Hepatocellular carcinoma cell; LLC-LM: Lewis lung carcinoma; CDD: Charged coupled device; MET: Mesenchymal-to-epithelial transition.

## Competing interests

The authors declare that they have no competing interests.

## Authors' contribution

JKP, SJJ and SWK performed animal experiments, immunoblot assay, PCR, statistical analysis. SP, SGH and WJK carried out animal tissue collection, immunohistochemistry and interpretation of the immunohistochemistry data. JHK designed experiments for bioluminescence imaging and HDU coordinated whole experiments. JKP, JHK and HDU prepared manuscript. All authors read and approved final manuscript.
